# Multiple Proteins of *Lacticaseibacillus rhamnosus* GG Are Involved in the Protection of Keratinocytes From the Toxic Effects of *Staphylococcus aureus*

**DOI:** 10.3389/fmicb.2022.875542

**Published:** 2022-05-11

**Authors:** Cecile El-Chami, Rawshan Choudhury, Walaa Mohammedsaeed, Andrew J. McBain, Veera Kainulainen, Sarah Lebeer, Reetta Satokari, Catherine A. O’Neill

**Affiliations:** ^1^Faculty of Biology, Medicine and Health, School of Biological Sciences, University of Manchester, Manchester, United Kingdom; ^2^Faculty of Biology, School of Health Sciences, Medicine and Health, University of Manchester, Manchester, United Kingdom; ^3^Faculty of Medicine, Human Microbiome Research Program, University of Helsinki, Helsinki, Finland; ^4^Department of Bioscience Engineering, University of Antwerp, Antwerp, Belgium

**Keywords:** probiotic, keratinocyte, *Lacticaseibacillus rhamnosus* GG, SpaC, moonlight proteins, *Staphylococcus aureus*

## Abstract

We have previously shown that lysates of *Lacticaseibacillus rhamnosus* GG confer protection to human keratinocytes against *Staphylococcus aureus. L. rhamnosus* GG inhibits the growth of *S. aureus* as well as competitively excludes and displaces the pathogen from keratinocytes. In this study, we have specifically investigated the anti-adhesive action. We have tested the hypothesis that this activity is due to quenching of *S. aureus* binding sites on keratinocytes by molecules within the *Lacticaseibacillus* lysate. Trypsinisation or heat treatment removed the protective effect of the lysate suggesting the involvement of proteins as effector molecules. Column separation of the lysate and analysis of discrete fractions in adhesion assays identified a fraction of moderate hydrophobicity that possessed all anti-adhesive functions. Immunoblotting demonstrated that this fraction contained the pilus protein, SpaC. Recombinant SpaC inhibited staphylococcal adhesion to keratinocytes in a dose-dependent manner and improved keratinocyte viability following challenge with viable *S. aureus*. However, SpaC did not confer the full anti-adhesive effects of the LGG lysate and excluded but did not displace *S. aureus* from keratinocytes. Further purification produced four protein-containing peaks (F1–F4). Of these, F4, which had the greatest column retention time, was the most efficacious in anti-staphylococcal adhesion and keratinocyte viability assays. Identification of proteins by mass spectrometry showed F4 to contain several known “moonlighting proteins”—i.e., with additional activities to the canonical function, including enolase, Triosephosphate isomerase (TPI), Glyceraldehyde 3 phosphate dehydrogenase (G3P) and Elongation factor TU (EF-Tu). Of these, only enolase and TPI inhibited *S. aureus* adhesion and protected keratinocytes viability in a dose-dependent manner. These data suggest that inhibition of staphylococcal binding by the *L. rhamnosus* GG lysate is mediated by SpaC and specific moonlight proteins.

## Introduction

Probiotics have been defined as “live micro-organisms that, when administered in adequate amounts, confer a health benefit to the host” (Hill et al., 2014). Usually members of the genera lactobacilli and bifidobacteria, probiotics have been reported to have beneficial effects such as prevention of antibiotic-associated diarrhoea (Vanderhoof et al., 1999) and reduction in the incidence of atopic disease (Kalliomäki et al., 2001). The mechanisms used by the bacteria to exert positive effects are varied and include inhibition of pathogens (Coconnier et al., 1993; Mukherjee and Ramesh, 2015), modulation of immune responses (Jespersen et al., 2015; Rossi et al., 2016) and enhancement of epithelial barrier functions (Laval et al., 2015; Simeoli et al., 2015). However, in general, the bacterial molecules underlying probiosis remain poorly characterised.

Due to the success of probiotics in the treatment of gut disorders, many authors have investigated whether lactic acid bacteria (LAB) hold any promise when used topically for the treatment or prevention of skin diseases (reviewed in Knackstedt et al., 2020). However, due to the potential problems associated with maintaining bacterial viability in topical formulations, use of LAB-derived molecules or their lysates have been investigated in a number of studies (Golkar et al., 2021; Nam et al., 2021). Although the term is still evolving, bacterially derived preparations are generally referred to as ‘postbiotics’ (Aguilar-Toalá et al., 2021; Salminen et al., 2021). We have been assessing a lysate of *Lacticaseibacillus rhamnosus* GG (LGG) as a novel topical treatment for skin in health and disease. Our previous *in vitro* data suggest that LGG lysate could be developed as a novel anti-infective agent to inhibit *Staphylococcus aureus* infection of skin. We have demonstrated that LGG lysate can protect epidermal keratinocytes from the toxic effects of *S. aureus* (Mohammedsaeed et al., 2014). Specifically, LGG lysate inhibits *S. aureus* adhesion to primary human keratinocytes and can both competitively exclude and displace *S. aureus* from keratinocyte binding sites. Additionally, LGG lysate can inhibit the growth of *S. aureus* (Mohammedsaeed et al., 2014). These two activities of LGG lead to an increase in keratinocyte survival in the presence of *S. aureus* (Mohammedsaeed et al., 2014).

In the current study, we have investigated the anti-adhesive effects of the LGG lysate against *S. aureus*. The mechanism by which LGG adheres to intestinal mucus has been shown previously to involve pili (Kankainen et al., 2009; Nishiyama et al., 2016) that promote retention of LGG in the gastrointestinal tract (Lebeer et al., 2012; Nishiyama et al., 2016). Pilus production in LGG is encoded by the *spaCBA* gene cluster, with the SpaC protein having been shown to be primarily responsible for the high mucus binding activity of LGG (Kankainen et al., 2009; Lebeer et al., 2012; Nishiyama et al., 2016). In our previous work, we showed that SpaC was at least part of the mechanism whereby live LGG inhibits binding of *S. aureus* to keratinocytes (Spacova et al., 2020). In other LAB, several other adhesins have been identified. Often, proteins of central metabolism, these are referred to as “moonlighting” proteins because of their dual functionality. However, the presence of moonlighting adhesins has been underexplored in LGG. We hypothesised that the protective activity of the LGG lysate may involve quenching of staphylococcal keratinocyte binding sites by molecules within the LGG lysate, which would otherwise be available for *S. aureus* binding. Thus, the aim of the present study was to investigate the molecules underlying LGG-mediated inhibition of *S. aureus* binding and protection of keratinocyte viability.

## Materials and Methods

### Bacterial Cell Culture

*Lacticaseibacillus rhamnosus* GG (ATCC 53103) was cultured anaerobically in Wilkins-Chalgren broth at 37°C, and *S. aureus* was cultured aerobically in Nutrient Broth (Oxoid) as previously described (Prince et al., 2012; Mohammedsaeed et al., 2014). Inhibition of staphylococcal growth using ‘spot on the lawn’ assay was performed as described in (Mohammedsaeed et al., 2014). LGG lysate was produced according to our previously published protocol (Mohammedsaeed et al., 2014). In some experiments investigating the involvement of proteins, the lysate was placed in a boiling water bath for 5 min or treated with trypsin (0.2%w/v) in Phosphate buffered saline for 1 h at 37°C to denature proteins.

### Fractionation of the *Lacticaseibacillus rhamnosus* GG Lysate

A 30 ml preparation of LGG lysate was adjusted to pH 5.8 using 0.1% Trifluoroacetic acid (TFA) and applied to a Strata XL column (pore size 100 μm, Phenomenex Ltd., Cheshire, United Kingdom). Bound proteins were eluted from the column in 60 ml of 90% methanol at United Kingdom) and the resulting sample (5 ml) was applied to a 5 ml Sep-Pak C18 cartridge (pore size 37–55 μm, Fischer Scientific, Loughborough, United Kingdom). Proteins were eluted using 5 ml aliquots of increasing concentrations of 10–70% (v/v) acetonitrile containing 0.1% (v/v) TFA solution. Each 5 ml fraction was collected into a separate tube and the eluted fractions were evaporated to remove the acetonitrile for 3 h in centrifugal evaporation system (Biotek, Bedfordshire, United Kingdom). The resulting 1 ml of each fraction was subjected to SDS-page analysis and stained with Instant Blue (Harston, Cambridgeshire, United Kingdom) to visualise the protein bands. The fractions were maintained at 4°C for further analysis in adhesion and viability assays. For increased concentration and purification, the most efficacious fractions were further separated by HPLC using a Jupiter 90A column (Phenomenex, Cheshire, United Kingdom) with a gradient of 10–99% acetonitrile applied over 50 min.

### Tandem Mass Spectrophotometric Analysis of Protein Fractions

Tandem Mass spectrometry (MS/MS) identification of proteins was conducted using the “gel top” method. Briefly, proteins were separated electrophoretically for 10 min at 150 V by SDS-PAGE and then stained using Instant Blue. Bands of interest were excised from the gel and dehydrated using acetonitrile followed by vacuum centrifugation. Dried gel pieces were reduced with 10 mM dithiothreitol and alkylated with 55 mM iodoacetamide. Gel pieces were then washed alternately with 25 mM ammonium bicarbonate followed by acetonitrile. This was repeated, and the gel pieces dried by vacuum centrifugation. Samples were digested with trypsin overnight at 37°C. Digested samples were analysed by LC–MS/MS using an UltiMate® 3000 Rapid Separation LC (RSLC, Dionex Corporation, Sunnyvale, CA) coupled to a LTQ Velos Pro (Thermo Fisher Scientific, Altrincham, United Kingdom) mass spectrometer. Peptide mixtures were separated using a gradient from 92% A (0.1% FA in water) and 8% B (0.1% FA in acetonitrile) to 33% B, in 44 min at 300 nl min^−1^, using a 75 mm x 250 μm i.d. 1.7 ·M BEH C18, analytical column (Waters). Peptides were selected for fragmentation automatically by data dependant analysis. Data produced were searched using Mascot data base search engine (Matrix Science United Kingdom). Data were validated using Scaffold (Proteome Software, Portland, OR).

### Growth of Primary Human Keratinocytes, Viability and Adhesion Assays

Primary human keratinocytes and associated adhesion and viability assays were performed exactly as previously (Mohammedsaeed et al., 2014).

### Production of Recombinant SpaC and Moonlight Proteins

Briefly, an expression plasmid construct (pKTH5319) encoding the *L. rhamnosus* GG (ATCC 53103) SpaC gene and an expression vector phi encoding the *L. rhamnosus* GG EF-Tu gene were created in the Manchester Protein Expression facility by Dr. Edward McKenzie. Expression vectors pETDuet-1 encoding the *L. rhamnosus* GG genes G3P, Enolase and TPI were created in GenScript Biotech (Netherlands). Recombinant SpaC, EF-Tu, G3P, Enolase and TPI hexa-histidine-tagged at the C-terminus were expressed in *One Shot™ BL21(DE3) pLysS Chemically Competent E.* coli. The expression of these recombinant proteins was then induced by addition of 1 mM Isopropyl β-D-1thiogalactopyranoside (IPTG) and cultures were incubated for 3 h at 37°C, 220 rpm. Cells were harvested by centrifugation at 4,000 rpm for 10 min, resuspended in a lysis buffer consisting of 25 mM Tris–HCl pH 8.0, 0.3 M NaCl, 1% (v/v) Triton X-100 and EDTA-free protease-inhibitor cocktail tablets (Roche Ltd., Sussex United Kingdom). The cell lysate was then centrifuged at 14,000x*g* for 30 min at 4°C and the cell-free lysate was loaded into a 1 ml HisPur™ Ni-NTA Spin Columns (Thermo Fisher Scientific, Altrincham, United Kingdom) that had previously been equilibrated with a buffer containing 25 mM Tris–HCl pH 8.0, 0.3 M NaCl and 5 mM imidazole. Columns were washed once with wash buffer containing 25 mM Tris–HCl pH 8.0, 0.3 M NaCl and 20 mM imidazole, 0.1% (v/v) Triton X-100 and 1:1000 protease-inhibitor cocktails and twice with wash buffer containing 25 mM Tris–HCl pH 8.0, 0.3 M NaCl and 50 mM imidazole. Resin-bound recombinant proteins were then eluted with buffer containing 25 mM Tris–HCl pH 8.0, 0.3 M NaCl and 250 mM imidazole. Recombinant proteins containing fractions were determined by SDS-PAGE.

### SDS-PAGE and Immunostaining

SpaC was detected using antiserum raised by immunising rabbit with recombinant SpaC expressed in *E. coli* as described in (Tytgat et al., 2016). HRP conjugated 6x-His tag monoclonal antibody (Invitrogen, United Kingdom) was used to target recombinant moonlight proteins. SDS-PAGE was performed as described in (Sultana et al., 2013).

### Statistical Analyses

All data are presented as the mean ± SEM of at least three independent experiments with triplicate samples within each independent experiment. All statistical tests were carried out using GraphPad Prism v9·0 software (GraphPad Software Inc., La Jolla, CA, United States). Data were analysed by One-way ANOVA followed by Bonferroni’s correction. Differences were considered statistically significant at a value of *p* < 0.05.

## Results

### Heat or Protease-Treated Lysates Do Not Protect Keratinocytes From the Effects of *Staphylococcus aureus*

**Figure 1:**
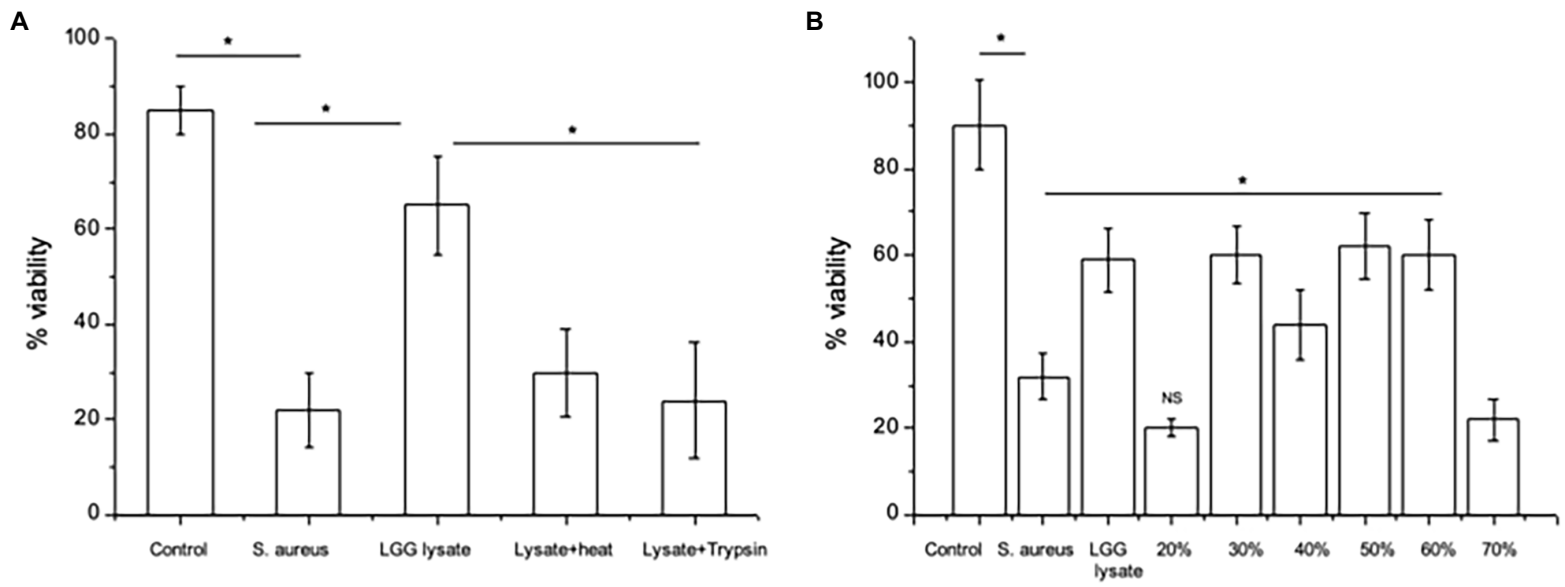
Proteins mediate the effects of LGG lysate against *Staphylococcus aureus*. **(A)** A combination of *S. aureus* and *Lacticaseibacillus rhamnosus* GG lysate (Lgg lys) resulted in a significantly higher percentage of viable keratinocytes than in monolayers infected with *S. aureus* alone (^*^p = 0.006). All data are compared to viability of untreated monolayers (Control). Heat (Lysate + heat) or protease treatment (Lysate + trypsin) destroyed the ability of the lysate to protect keratinocytes from *S. aureus*. **(B)** When the lysate was fractionated and proteins eluted in 10–70% acetonitrile, proteins with efficacy against *S. aureus* were contained in fractions 30–60%. Data were generated using primary human keratinocytes from three different donors and are presented as the mean ± SEM, *n* = 3. ns = Non-significant.

We began by determining the nature of the effector molecules within the LGG lysate mediating its anti-adhesive effects on *S. aureus*. To this end, we treated the lysate with heat or protease and then investigated its ability to protect keratinocyte monolayer viability in the presence of *S. aureus*. In agreement with our previous findings (Mohammedsaeed et al., 2014), only *c*. 20–30% of the keratinocyte monolayer was viable following 24 h incubation with *S. aureus*, while in the presence of LGG lysate, monolayer viability increased to *c.* 65%. However, heat or protease-treated lysate did not protect monolayer viability (Figure 1A), suggesting that proteins within the lysate are the effector molecules.

The LGG lysate was then subjected to partial fractionation using a reverse-phase column and the proteins were eluted in a gradient of 10–70% acetonitrile (Supplementary Figure S1). The ability of proteins eluting in each fraction to protect keratinocyte viability was investigated. Proteins eluting in 30–60% acetonitrile were able to protect keratinocyte from the effects of *S. aureus*. However, proteins eluting in other fractions did not (Figure 1B).

### Proteins Eluting in the 50% Acetonitrile Fraction Efficiently Exclude and Displace *Staphylococcus aureus* From Keratinocytes

**Figure 2:**
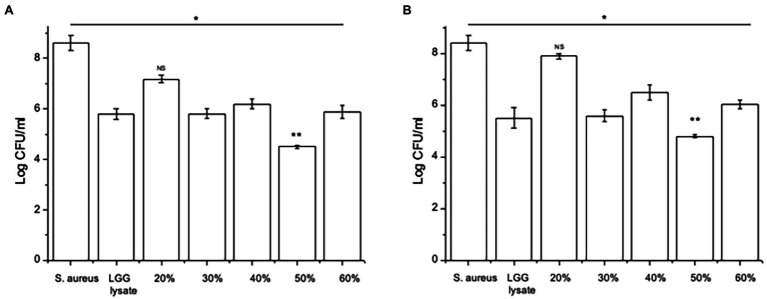
Specific fractions inhibit staphylococcal adhesion to keratinocytes. **(A)** Keratinocytes pre-treated with *L. rhamnosus* GG lysate had significantly fewer adherent *Staphylococci* compared to cells infected with *S. aureus* alone. The adhesion of the pathogen to keratinocytes was significantly lower in cultures treated with fractions eluting in 30%, 40%, 50% and 60% acetonitrile (*p = 0.01, *p = 0.016, **p = 0.012, and *p = 0.015, respectively). **(B)** The same fractions were also efficacious when added to keratinocytes 2 h after incubation with pathogen (*p = 0.034, *p = 0.035, **p = 0.01, and v*p* = 0.033 for 30%, 40%, 50%, and 60%, respectively). Data are presented as the mean ± SEM, *n* = 3. ns = Non-significant.

Since we have shown before that there is a link between inhibition of *S. aureus* adhesion and protection of keratinocyte viability (Prince et al., 2012; Mohammedsaeed et al., 2014), we next tested the ability of the 30–60% fractions of the lysate to exclude or displace *S. aureus* from keratinocyte binding sites. The data in Figure 2 show that proteins contained in the fraction eluting in 50% acetonitrile (50% fraction) are the most efficacious at both exclusion (Figure 2A) and displacement (Figure 2B) of *S. aureus*. Importantly, when tested this fraction in a ‘spot on the lawn’ assay, it had minimal activity against *S. aureus* growth compared with other fractions that conferred protection to keratinocytes (Table 1). Therefore, we could be confident that the ability of the 50% fraction to protect keratinocyte viability was due to its anti-adhesive properties and not any effects on pathogen growth.

**Table 1:** The Inhibition of *Staphylococcus aureus* growth by whole and fractionated LGG lysate.

Treatment	Diameter of zone of inhibition (mm, *n* = 3)
Whole LGG lysate	14+/− 1.6
20% fraction	0
30% fraction	5+/− 0.8
40% fraction	0
50% fraction	0
60% fraction	10+/−1.7
99% acetonitrile	0

*Data shown are the mean and SEM of at least three separate experiments.*

### The SpaC Pilus Protein Inhibits Adhesion of *Staphylococcus aureus* to Keratinocytes

**Figure 3:**
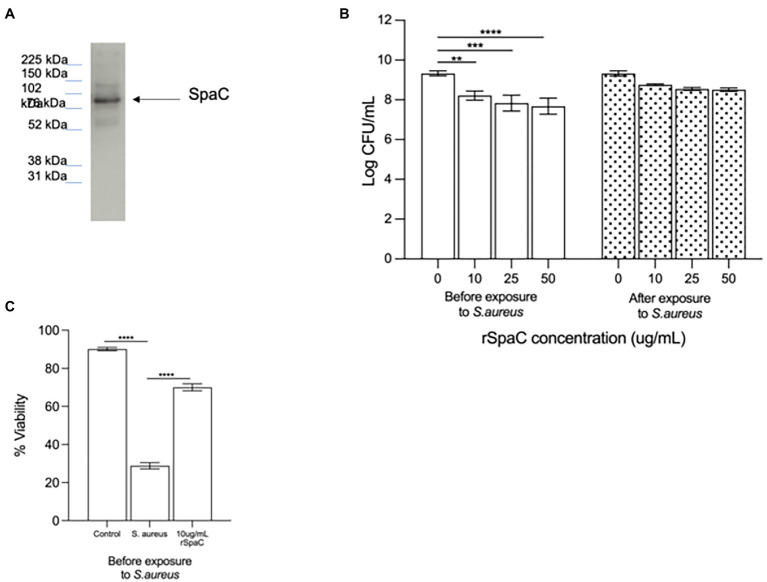
The pilus protein SpaC is involved in the anti-adhesive function of the lysate. **(A)** Immunoblotting with specific anti-SpaC serum demonstrated the presence of the SpaC protein in the 50% fraction. **(B)** Addition of recombinant SpaC (rSpaC) prior to addition of the pathogen inhibited staphylococcal adhesion to keratinocytes in a dose-dependent manner (**p = 0.003, ***p = 0.0003, and ****p < 0.0001 for 10, 25, and 50 μg/mL, respectively, *n* = 5). However, when added subsequent to exposure of cells to *Staphylococcus aureus*, rSpaC did not inhibit adhesion (*n* = 3). **(C)** rSpaC at 10 μg/ml afforded significant protection to keratinocyte monolayer viability in the presence of *S. aureus* when added prior to the addition of the pathogen (*p* = 0.0033). When added subsequent to exposure of cells to *S. aureus*, rSpaC did not protect keratinocyte viability. Data are presented as the mean ± SEM. ns = Non-significant.

Since we have previously shown that the protein SpaC is part of the mechanism by which live LGG inhibits adhesion of *S. aureus* to live keratinocytes (Spacova et al., 2020), we hypothesised that this protein may also be part of the inhibitory action of the lysate and chose to investigate this as a starting point for our study. To understand the involvement of SpaC in anti-adhesive processes, the 50% fraction was subjected to immunoblotting using an anti-SpaC antibody. This produced a single band, at the correct molecular weight for SpaC, suggesting that the 50% fraction of the lysate contains this protein (Figure 3A). To confirm the involvement of SpaC as an anti-*S. aureus* adhesion mechanism, we produced recombinant SpaC (rSpaC) and compared its anti-adhesive activity against that of the crude lysate. Pre-treatment of keratinocyte monolayers with rSpaC inhibited adhesion of *S. aureus* to keratinocytes in a dose-dependent manner (Figure 3B). However, rSpaC did not reduce staphylococcal adhesion when it was added to keratinocytes after addition of the pathogen (Figure 3B). By contrast, 50 μg/ml of a control protein, bovine serum albumin did not inhibit staphylococcal adhesion to keratinocytes whether added to keratinocytes either before or after the pathogen (Supplementary Figure S2).

To determine whether our previously established link between anti-adhesive effects of the whole LGG lysate and protection of keratinocyte viability holds true for SpaC, we tested whether rSpaC could protect keratinocyte monolayers from the toxic effects of *S. aureus*. Our data showed that 10 μg/ml of rSpaC protect keratinocytes and significantly improve their viability when exposed to *S. aureus* (Figure 3C). However, rSpaC could not protect keratinocytes when added after the pathogen (data not shown).

### The 50% Acetonitrile Fraction Contains Additional Potential Anti-adhesive Proteins

**Figure 4:**
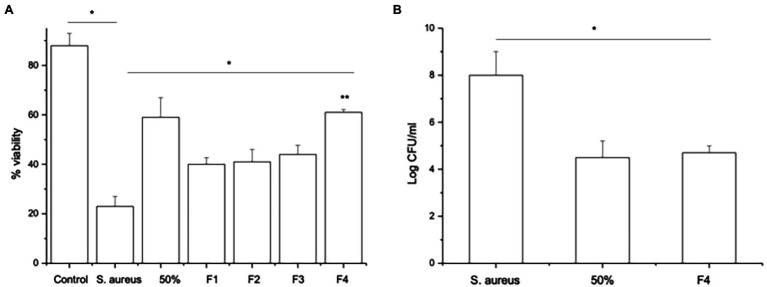
Fraction F4 contains all the anti-adhesive effects of the LGG lysate. **(A)** The viability of keratinocytes infected with *S.aureus* and treated either with 50% acetonitrile fraction (50%) or with *S. aureus* and the F4 HPLC fraction (F4) significantly increased compared to keratinocytes infected with *S. aureus* alone (*p = 0.04 and **p = 0.003, respectively). However, the other fractions (F1, F2, and F3) afforded less protection to keratinocytes than cells treated with F4 (*p = 0.02). **(B)** Cells exposed to *S. aureus* for 2 h and then treated with either 50% acetonitrile fraction (50%) or HPLC fraction F4 (F4) have significantly fewer *staphylococci* adhering to the cells than cells infected with *S. aureus* alone (*p = 0.04). Data are presented as the mean ± SEM., *n* = 3.

Since SpaC does not contain all the anti-adhesive properties of the whole lysate, i.e., it excludes but does not displace *S. aureus*, we considered the possibility that other proteins in the LGG lysate may also impact upon adhesion of staphylococci to keratinocytes. Therefore, we performed tandem mass spectrometry (MS/MS) analysis of the proteins contained within the 50% fraction of the LGG lysate. The data are summarised in Table 2. To further concentrate and identify proteins of interest, we performed a further round of purification of the 50% fraction using Reverse-Phase HPLC and proteins were eluted from a C18 reverse-phase column using a gradient of 0–100% acetonitrile. The concentrated fractions were collected based on ultraviolet absorption at 215 nm and 4 specific peaks containing proteins were collected at between 21 and 32 min. of elution. These peaks, named F1–4 (to differentiate their retention times on the column; Supplementary Figure S3), were used in both staphylococcal adhesion assays and keratinocyte viability assays. The proteins contained within F4 were the most efficacious in both assays (Figures 4A,B). Hence, F4 was subjected to analysis both by gel electrophoresis and MS/MS analysis. The proteins contained within F4 are shown in Table 3. The proteins highlighted in bold in Table 3, elongation factor Tu, (EF-Tu) glyceraldehyde-3-phosphate dehydrogenase (G3P), enolase and triosephosphate isomerase (TPI), are common to both the 50% acetonitrile fraction and the F4 fraction. These proteins are likely to be major constituents of F4 because the most abundant proteins in the F4 fraction (as judged by electrophoresis) correspond to the known molecular weights of these proteins (Supplementary Figure S3).

**Table 2:** Proteins identified by tandem mass spectrometry in the 50% acetonitrile fraction of the LGG lysate.

Protein	Molecular weight (kDa)
UDP-glucose 4 epimerase	90
B-galactosidase chain D	33
30S Ribosomal protein S7	95
Acyl carrier protein	9
**Glyceraldehyde-3 phosphate dehydrogenase**	**36**
**Elongation factor TU**	**44**
**Triosephosphate isomerase**	**27**
50S ribosomal protein S11	15
Dihydroxyacetone kinase	21
50S ribosomal protein L22	13
Asparaginyl tRNA synthetase	50
**Enolase**	**47**
GMP synthase	58
UPFO342 protein LRH	13
30S ribosomal protein S5	32
Glucose 1 phosphate thymidylyltransferase	75
B-galactosidase chain D	33
DNA-directed RNA polymerase alpha subunit	38
Phosphoribosylpyrophosphate synthetase	25
Phosphoglycerate mutase	46
Aspartyl tRNA synthetase	64
M29 family amonopeptidase	21
Glycine cleavage systemH	11
50S ribosomal protein	13
UPF0342 protein	13

*These proteins were consistently found in n* = *3 column fractionations. The bolded text highlights proteins with molecular weights corresponding to abundant proteins in the sample as judged by SDS-PAGE*.

**Table 3:** Proteins identified by tandem mass spectrometry in the F4 fraction of the lysate.

Protein	Molecular weight (kDa)
Acyl carrier protein	9
**Glyceraldehyde-3 phosphate dehydrogenase**	**36**
**Elongation factor TU**	**43**
Transcription elongation factor greA	23
Phosphopentomutase	43
**Triosephosphate isomerase**	**27**
50S ribosomal protein S11	15
Dihydroxyacetone kinase	21
50s Ribosomal protein	15
Asparaginyl tRNA synthetase	50
**enolase**	**47**
UPF0342 protein	13
50S ribosomal protein L22	13

*The bolded text indicates proteins with molecular weights corresponding to abundant proteins in the fraction as judged by SDS-PAGE*.

### Specific Moonlighting Proteins, G3P and Enolase Inhibit Adhesion of *Staphylococcus aureus* to Keratinocytes

The moonlighting proteins EF-Tu, G3P, enolase and TPI have been previously associated with the host-probiotic interactions (Kainulainen and Korhonen, 2014). In order to understand their involvement in anti-adhesive processes in keratinocytes, recombinant moonlight proteins were produced. Immunoblotting targeting His-Tagged moonlighting proteins revealed bands that correspond to the known molecular weights of these proteins (Supplementary Figure S4).

**Figure 5:**
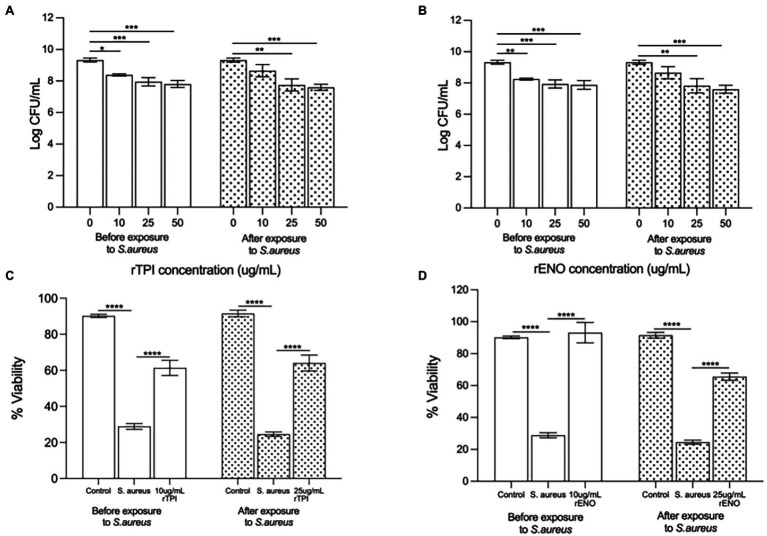
**(A)** Recombinant TPI (rTPI) and **(B)** recombinant ENO (rENO) added prior to addition of the pathogen inhibited staphylococcal adhesion to keratinocytes in a dose dependent manner (*p = 0.0238, *p* = 0.0107 and *p* = 0.0030 for 10, 25 and 50 ug/mL of rTPI respectively and **p = 0.0063, ***p = 0.0095 and ***p = 0.0052 for 10, 25 and 50 ug/mL of rENO respectively). However, when added subsequent to exposure of cells to *S. aureus*, **(A)** rTPI and **(B)** rENO only 25 and 50 ug/mL inhibited *S.aureus* adhesion (**p = 0.0007 and ***p < 0.0001 for 25 and 50 ug/mL of rTPI respectively and **p = 0.0012 and ***p < 0.001 for 25 and 50 ug/mL of rENO respectively). Recombinant TPI **(C)** and rENO **(D)** at 10 µg/ml afforded significant protection to keratinocyte monolayer viability in the presence of *S. aureus* when added prior to the addition of the pathogen (****p < 0.0001). When added subsequent to exposure of cells to *S. aureus*, rTPI **(C)** and rENO **(D)** at 25 µg/ml afforded significant protection to keratinocyte monolayer viability in the presence of *S. aureus* (****p < 0.0001). Data are presented as the mean ± SEM *n* = 3.

Pre-treatment of keratinocyte monolayers with recombinant TPI (rTPI; Figure 5A) and recombinant ENO (rENO; Figure 5B) at 10, 25 or 50 μg/ml inhibited adhesion *S. aureus* to keratinocytes in a dose-dependent manner. Treatment of keratinocyte monolayers with 25 and 50 (but not 10) μg/mL rTPI (Figure 5A) and rENO (Figure 5B) for 2 h following infection with *S. aureus* significantly inhibited the pathogen adhesion. In agreement with this data, the lowest concentrations of rENO and rTPI that inhibited adhesion, also increased the viability of keratinocyte monolayers challenged with *S. aureus*. Thus, treatment of keratinocytes, prior to exposure to *S. aureus*, with 10 μg/mL of rTPI (Figure 5C) and rENO (Figure 5D) was found to protect the monolayer from the toxic effect of the pathogen in a viability assay. However, when applied after exposure to *S. aureus*, 25 μg/mL rTPI (Figure 5C) and rENO (Figure 5D) protected keratinocytes and significantly improved monolayer viability.

On the other hand, pre-treatment or post-treatment with recombinant G3P (rG3P; Supplementary Figure S5A) and recombinant EF-Tu (rEF-Tu; Supplementary Figure S5B) had no effect on the adhesion of *S. aureus* to keratinocytes monolayers and did not protect viability (Supplementary Figures S5C,D).

## Discussion

We have been studying the potential of LGG cell-free lysates as a topical therapy targeted at prevention/treatment of *S. aureus* skin infection. For topical applications, a cell-free lysate has many advantages over the use of viable bacteria. Importantly, the safety concerns surrounding use of live bacteria on the skin would be negated as would be the potential problems associated with formulating live bacteria. Such bacterial preparations have recently been referred to as “postbiotics” which are defined as “a preparation of inanimate microorganisms and/or their components that confers a health benefit on the host” (Aguilar-Toalá et al., 2021; Salminen et al., 2021). However, if potential postbiotics like LGG lysate are to fulfil their potential as topical therapeutic agents, an understanding of the effector molecules mediating their effects is a pre-requisite. We have therefore investigated the molecules mediating the anti-adhesive effects of LGG against *S. aureus*. The efficacious molecules are apparently proteins because heat denaturation or protease treatment completely destroyed the activity of the lysate against *S. aureus*. However, the possibility that other molecules such as sugars on the surface of LGG may also be important to the anti-adhesive action of the lysate cannot be completely excluded. Indeed, exopolysaccharides have been shown to be important for LGG binding to the host in the intestine (Ruas-Madiedo et al., 2006).

A number of protein adhesins have been previously identified in lactobacilli (Pancholi and Fischetti, 1992; Edelman et al., 2012; Kainulainen and Korhonen, 2014). Of these, the involvement of the pilus protein SpaC as a mucus binding protein has been shown in a number of studies (Kankainen et al., 2009; Lebeer et al., 2012). Our present work shows that SpaC may also be involved in the mechanism by which LGG inhibits *S. aureus* adhesion. This is suggested by a number of observations: firstly, fractionation of the lysate and analysis of the fractions show the most efficacious fraction to contain SpaC; secondly, recombinant SpaC inhibits *S. aureus* adhesion in a dose-dependent manner; and lastly, the toxic effects of *S. aureus* on keratinocyte viability are negated by SpaC, but not a control protein, BSA.

Overall, these data are consistent with a conclusion that SpaC is involved in the mechanism by which LGG lysate inhibits the adhesion of *S. aureus* to keratinocytes. Indeed, in other work, SpaC was shown to be intimately involved in the mechanism by which live LGG protects keratinocytes from the effects of *S. aureus* (Spacova et al., 2020). However, the previous study only investigated the role of SpaC when live LGG was added to keratinocytes at the same time as the pathogen (Spacova et al., 2020). In the present study, using an LGG lysate, SpaC is almost certainly not the only protein involved. Of note in this regard is the observation that SpaC excludes but does not displace *S. aureus* from keratinocyte binding sites, whereas the whole LGG lysate possesses both activities. This suggests that other proteins are also involved in the full anti-adhesive activities of the lysate. Indeed, several other proteins were found in the 50% fraction and this fraction contains all the anti-adhesive activities of the lysate. Crucially, the 50% fraction does not inhibit the growth of *S. aureus* thus suggesting that adhesive proteins are likely to be the only molecules in this fraction responsible for its observed effects. The proteins contained within the 50% fraction were further concentrated by HPLC into a fraction (F4) which showed the highest efficacy in both adhesion and viability assays. The most abundant proteins in this F4 fraction are likely to be elongation factor Tu (EF-TU), glyceraldehyde-3-phosphate dehydrogenase (G3P), enolase and triosephosphate isomerase (TPI), because the major components of the fraction (as judged by gel electrophoresis) were proteins of the same molecular weights as these. Interestingly, SpaC must be a low abundance protein in both the 50% and F4 fractions because gel-top Tandem Mass Spectrometry identification of proteins did not detect it. Indeed, this technique is known to be reliable only for identification of abundant proteins (D. Knight, University of Manchester protein sequencing unit, personal communication). However, SpaC was shown to be present by Western blotting (which has an amplification effect and so is more sensitive than mass spectrometry) suggesting it is present in the fraction and hence may be part of the anti-adhesive mechanism. SpaC is part of the heteropolymeric SpaCBA pili that are complex structures. It is known that low protein expression can still result in high adhesive ability due to the "zipperlike” mechanism of adhesion’ (Segers and Lebeer, 2014). However, the mechanism of inhibiting *S. aureus* binding to keratinocytes appears to additionally involve different proteins probably because it is a completely different system.

The proteins EF-TU, G3P, enolase, and TPI have been previously reported to be important for adhesive function in several species of lactobacilli (Pancholi and Fischetti, 1992; Granato et al., 2004; Ramiah et al., 2008; Kainulainen et al., 2012; Dhanani and Bagchi, 2013; Glenting et al., 2013; Spurbeck et al., 2015; Jeffery, 2018). All these proteins have been previously described as so-called “moonlighting proteins”, i.e., proteins with an ability to perform functions unrelated to the canonical function ascribed to the protein (Kainulainen and Korhonen, 2014). For example, G3P is an intracellular enzyme central to glycolysis. However, it is found as a cell surface adhesion protein on several prokaryotes including *L. plantarum* and *L. crispatus* (Pancholi and Fischetti, 1992; Kainulainen et al., 2012). TPI, another glycolytic enzyme, has been shown to be involved in competitive exclusion and displacement of *Clostridium sporogenes* and *Enterococcus faecalis* from Caco-2 cells by *L. plantarum* (Ramiah et al., 2008). EF-Tu is involved in protein translation but is found at the cell surface as an adhesin mediating attachment of lactobacilli to mucins (Granato et al., 2004; Dhanani and Bagchi, 2013). Many of these moonlighting proteins have been shown to mediate bacterial adhesion to eukaryotic cells by binding to specific eukaryotic proteins such as fibronectin (Muñoz-Provencio et al., 2011; Salzillo et al., 2015). Evidence has demonstrated that enolase, EF-TU and G3P to be cell surface proteins of LGG (Espino et al., 2015) as well as having their usual cytoplasmic location. Such dual localisation is usually suggestive of moonlighting function (Kainulainen and Korhonen, 2014). In the current study, we show for the first time an anti-staphylococcal adhesive function of the moonlighting proteins TPI and enolase in *L. rhamnosus* GG. Interestingly, G3P and EF-Tu that did not inhibit *S. aureus* adhesion also did not protect keratinocytes demonstrating that these effects are due to specific proteins and not a random effect of protein present within the assay. These data suggest that one mechanism by which the lysate protects form the *S. aureus*-induced cell death of keratinocytes involves the ability of SpaC, TPI and enolase proteins to prevent the adhesion of *S. aureus* to the keratinocyte surface. Of note is the small but significant effects on adhesion which appear to be sufficient for a large effect on viability of keratinocytes. This may be due to reduced bacterial load. Potentially a small reduction in load may be sufficient to render the keratinocytes more capable of resisting the toxic effects of *S. aureus* perhaps by processes such as production of antimicrobial peptides (Mohanty et al., 2017; Abu-Humaidan et al., 2018).

Both the concentration and timing of LGG protein addition to the keratinocytes monolayers appear to be critical to its protective effect. When SpaC, TPI and enolase were added before *S. aureus*, keratinocytes were protected from the toxic effects of the pathogen in a dose-dependent manner. However, only the higher concentration (25 and 50 μg/mL) of TPI and enolase were able to protect and reduce keratinocyte cell death levels when added after the pathogen. Taken together our data suggest that various LGG lysate protein components confer different mechanisms of protection involving exclusion and displacement of *S. aureus* from keratinocyte binding sites.

In summary, we suggest that the overall anti-adhesive function of LGG against *S. aureus* may be facilitated by a number of proteins including SpaC, and the moonlighting proteins TPI and enolase, although others may also be involved. We hypothesise that a number of proteins, including the ones identified, act together to inhibit *S. aureus* adhesion although the exact contribution of each remains to be established.

## Data Availability Statement

The original contributions presented in the study are included in the article/Supplementary Material, further inquiries can be directed to the corresponding author.

## Author Contributions

RC and CE-C designed and conducted the experiments, and performed data analysis on SpaC and moonlighting proteins. CE-C drafted the manuscript with support from CO. WM performed some additional viability assays and lysate fractionation. VK produced the SpaC antibody. AM, SL, and RS assisted with theoretical interpretation. CO conceived the project, supervised the work and oversaw the production of the manuscript. All authors contributed to the article and approved the submitted version.

## Funding

The authors declare that this study received funding from SkinBiotherapeutics PLC. The funder was not involved in the study design, collection, analysis, interpretation of data, the writing of this article or the decision to submit it for publication.

## Conflict of Interest

CO and AM have equity in SkinBioTherapeutics. However, the work described in this manuscript is purely of academic interest.

The remaining authors declare that the research was conducted in the absence of any commercial or financial relationships that could be construed as a potential conflict of interest.

## Publisher’s Note

All claims expressed in this article are solely those of the authors and do not necessarily represent those of their affiliated organizations, or those of the publisher, the editors and the reviewers. Any product that may be evaluated in this article, or claim that may be made by its manufacturer, is not guaranteed or endorsed by the publisher.
