# Supplementary figures and images for: Multiple Proteins of Lacticaseibacillus rhamnosus GG Are Involved in the Protection of Keratinocytes From the Toxic Effects of Staphylococcus aureus
Source: Front Microbiol. 2022 May 11;13:875542. doi: 10.3389/fmicb.2022.875542 (PMC9134637; doi:10.3389/fmicb.2022.875542)

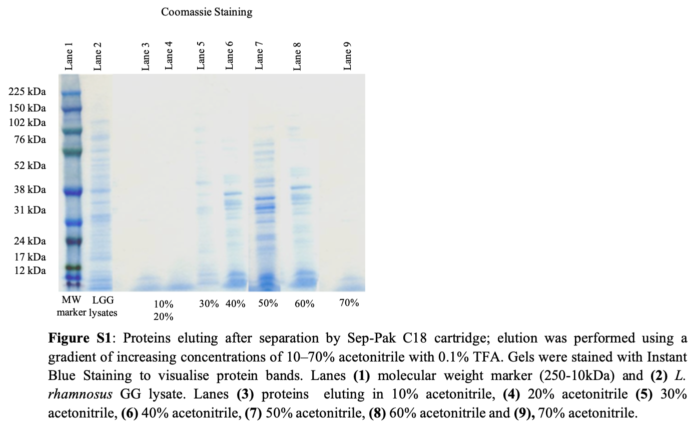

Supplement: Supplementary file 1 [file Image_1.TIF]

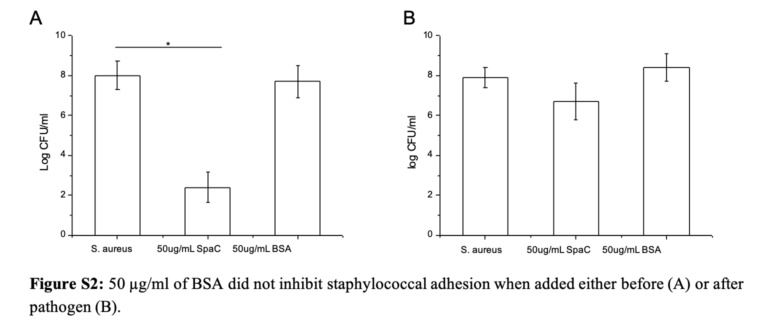

Supplement: Supplementary file 2 [file Image_2.TIF]

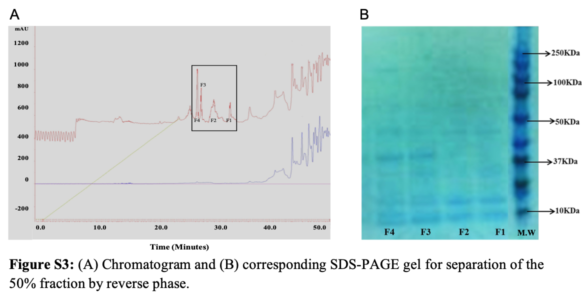

Supplement: Supplementary file 3 [file Image_3.TIF]

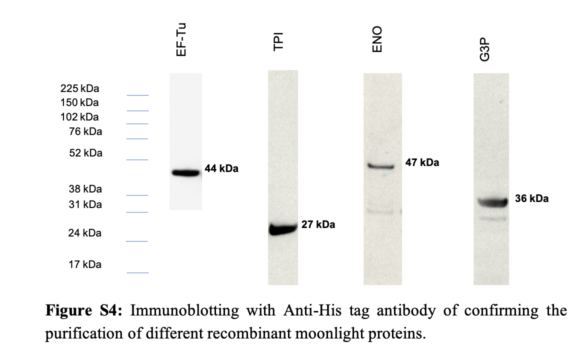

Supplement: Supplementary file 4 [file Image_4.TIF]

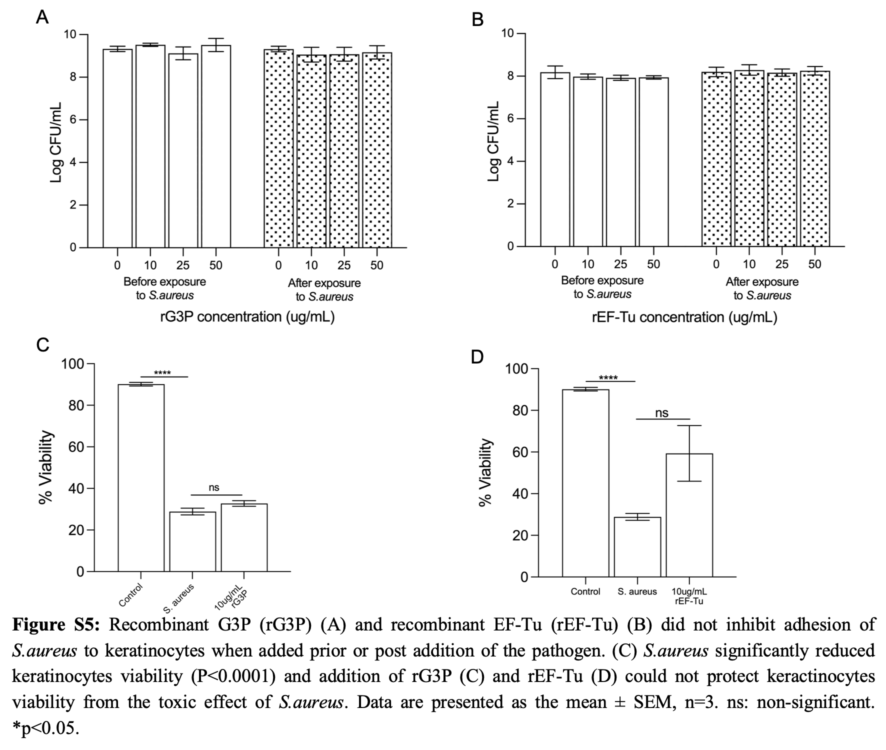

Supplement: Supplementary file 5 [file Image_5.TIF]
